# In Vivo Anti-Tumor Effects of Citral on 4T1 Breast Cancer Cells via Induction of Apoptosis and Downregulation of Aldehyde Dehydrogenase Activity

**DOI:** 10.3390/molecules24183241

**Published:** 2019-09-05

**Authors:** Siyamak Ebrahimi Nigjeh, Swee Keong Yeap, Norshariza Nordin, Heshu Rahman, Rozita Rosli

**Affiliations:** 1 Institute of Bioscience, Universiti Putra Malaysia, Serdang 43400, Selangor, Malaysia; 2 Faculty of Life Science and Biotechnology, Shahid Beheshti University, Daneshjou Boulevard, Tehran 1983969411, Iran; 3 Department of Medical Genetics, Tehran University of Medical Sciences, Poursina street, Tehran 1366736511, Iran; 4 China-ASEAN College of Marine Sciences, Xiamen University Malaysia, Jalan Sunsuria, Bandar Sunsuria, Sepang 43900, Selangor, Malaysia; 5 Faculty of Medicine and Health Sciences, Universiti Putra Malaysia, Serdang 43400, Selangor, Malaysia; 6 Department of Medical Laboratory Sciences and Technology, College of Health Sciences, Komar University of Science and Technology, Chaq Chaq Qularaese, Sarchinar District, Sulaimani 334, Iraq; 7 Department of Clinical and Internal Medicine, College of Veterinary Medicine, University of Sulaimani, Sulaimani 334, Iraq

**Keywords:** breast cancer, tumorigenicity, ALDH, citral

## Abstract

Breast cancer is the most commonly diagnosed cancer and the leading cause of cancer death among females globally. The tumorigenic activities of cancer cells such as aldehyde dehydrogenase (ALDH) activity and differentiation have contributed to relapse and eventual mortality in breast cancer. Thus, current drug discovery research is focused on targeting breast cancer cells with ALDH activity and their capacity to form secondary tumors. Citral (3,7-dimethyl-2,6-octadienal), from lemon grass (*Cymbopogon* *citrates*), has been previously reported to have a cytotoxic effect on breast cancer cells. Hence, this study was conducted to evaluate the in vivo effect of citral in targeting ALDH activity of breast cancer cells. BALB/c mice were challenged with 4T1 breast cancer cells followed by daily oral feeding of 50 mg/kg citral or distilled water for two weeks. The population of ALDH^+^ tumor cells and their capacity to form secondary tumors in both untreated and citral treated 4T1 challenged mice were assessed by Aldefluor assay and tumor growth upon cell reimplantation in normal mice, respectively. Citral treatment reduced the size and number of cells with ALDH^+^ activity of the tumors in 4T1-challenged BALB/c mice. Moreover, citral-treated mice were also observed with smaller tumor size and delayed tumorigenicity after reimplantation of the primary tumor cells into normal mice. These findings support the antitumor effect of citral in targeting ALDH^+^ cells and tumor recurrence in breast cancer cells.

## 1. Introduction

Breast cancer remains a leading cause of death in women worldwide. Despite recent advances in diagnosis and surgical techniques, as well as local and systematic adjuvant therapies, the rate of cancer recurrence that subsequently leads to mortality still remains high [1]. Many patients develop tumor recurrence and eventually succumb to the disease due to chemoresistance. Hence, understanding the mechanisms mediating survival of drug-resistant subpopulations of breast cancer cells is necessary to improve outcomes of this disease. Aldehyde dehydrogenase (ALDH^+^) cancer cells, a cancer stem-like cell, were found responsible for cancer related mortality and also for the failure of conventional cancer treatment therapies [2]. Therefore, understanding the pathogenesis of these cells with the capacity to form secondary tumors is key to ensuring the successful development of novel and more effective strategies in cancer management [3]. The ability of phytochemicals to inhibit tumor formation both in vitro and in vivo is well documented [4]. Many of these compounds have antioxidant, antiproliferative, and proapoptotic effects on various types of cancers [5]. These phytochemicals are commonly found in many food products and, thus, are well tolerated by humans. Furthermore, phytochemicals can be taken on a long-term basis to either prevent primary tumor formation or tumor recurrence [6]. For example, Fu et al. [7] reported that 100 mg/kg of resveratrol inhibited tumor growth in mice by suppressing the Wnt/β-catenin signaling pathway. Moreover, Kakarala et al. [8]. demonstrated that curcumin and piperine are able to modulate the capability of ALDH^+^ cells (marker for breast cancer stemness) and also inhibit Wnt signaling. Citral is an aldehyde compound found in *Cymbopogon citratus* (lemon grass), which has been shown to exert antiproliferative effects in MCF-7 and MDA-MB231 breast cancer cells through induction of apoptosis [9,10]. In addition, citral was also reported to inhibit the cancer stem-like cells of highly metastatic triple-negative MDA-MB231 breast cancer cells through suppression of ALDH activity [11,12]. Thus, it is valuable to evaluate the inhibitory effect of ALDH activity by citral on the triple-negative breast cancer cells in vivo. Triple-negative 4T1 murine breast cancer cells are highly tumorigenic and metastatic, commonly used as an animal model to represent human aggressive mammary cancer [13]. The present study evaluated the antitumor effect of citral by targeting the ALDH population of breast cancer cells and their capacity to form secondary tumors using a 4T1 breast cancer cell mice model. The outcome of this study may enable us to discover additional preventive and therapeutic strategies by using citral in cancer management to prevent cancer relapse and improve cancer patient survival. 

## 2. Materials and Methods

### 2.1. Cell Line and Reagents

The mouse triple-negative 4T1 breast cancer cell line was obtained from the American Type Culture Collection (ATCC). Citral was purchased from Sigma Aldrich, St. Louis, MO, USA. The stock solution was prepared in 5% dimethyl sulfoxide (DMSO) (Sigma Aldrich, Louis, MO, USA) and stored at −20 °C until further use. The β-catenin antibody was purchased from Cell Signaling Technology (Danvers, MA, USA).

### 2.2. Animal and Ethics Statement

Thirty-two adult female BALB/c mice aged 4 weeks were purchased from the animal house at the Faculty of Medicine and Health Sciences, Universiti Putra Malaysia (UPM). The mice were acclimatized to the laboratory environment at 24 ± 1 °C under a 12 hours’ dark–light cycle for 7 days, before commencement of the experiments. 

A previous study reported that mice treated with 100 mg/kg body weight of citral were observed with abnormalities. On the other hand, concentration between 40 to 80 mg/kg of citral showed antitumor effects on 4T1 challenged mice [14]. The pilot study (total *n* = 6, each concentration *n* = 2) using 40, 50, and 70 mg/kg of citral showed that 50 mg/kg of citral treatment was the minimum concentration that was able to reduce 50% of tumor volume after 21 days of treatment. Thus, 50 mg/kg of citral, which is half of the toxic dosage, was evaluated for the subchronic toxicity using normal mice and antitumor effect using 4T1 challenged mice. In addition, this concentration also falls under the effective dosage reported by the previous preliminary study [14]. For the subchronic toxicity (total *n* = 10) observation, 5 normal mice were orally fed 50 mg/kg for 28 days while 5 mice fed phosphate buffer saline (PBS) were kept as normal control. Clinical abnormalities were observed twice each day (morning and afternoon) for the onset of clinical or toxicological symptoms. Mortality, if any, signs of toxicity, body weight, food consumption, and gross findings were observed over a period of 28 days post-treatment. For the antitumor study (total *n* = 12), 6 mice were used in the induction of tumor and 6 mice were used as negative controls. The mice were provided pellets and water *ad-libitum* during the period of study at the Comparative Medicine and Technology Unit (COMeT) animal facility. The Institutional Animal Care and Use Committee approval was obtained: UPM/IACUC/AUP-R083/2013.

### 2.3. Breast Tumor Formation in Mice

The 4T1 cells (2 × 10^6^) were mixed with 200 µL of Matrigel (BD Biosciences, Franklin Lakes, NJ, USA), and injected in the lower fourth inguinal mammary fat pads of 5-week-old female BALB/c mice, as previously described [15]. Group 1 (*n* = 5) comprised of mice induced to develop breast tumor using 4T1 cells (2 × 10^6^) fed with PBS served as the breast tumor control, and group 2 (*n* = 6) comprised of mice induced to develop breast tumor using 4T1 cells (2 × 10^6^), treated orally with 50 mg/kg body weight each of citral for 28 days (Sigma Aldrich, Louis, MO, USA), respectively. Animals were observed for clinical abnormalities twice each day (morning and afternoon) for the onset of clinical or toxicological symptoms. Mortality, if any, signs of toxicity, body weight, food consumption, and gross findings were observed over a period of 28 days post-treatment. Individual body weights were obtained for the test animals prior to dosing on day 0, and on days 7, 14, 21, and 28. Tumors were measured using a calliper and the tumor volumes were calculated using the formula V = 1/2 (width^2^ × length).

### 2.4. Histopathology

Collected breast tumor and lung samples were cut into sections of about 0.5 cm^2^ sizes and fixed in 10% formalin for at least 48 h. The fixed samples were placed in plastic cassettes and dehydrated using an automated tissue processor (Leica ASP300, Leica, Wetzlar, Germany). The processed tissues were embedded in paraffin wax (Leica EG1160, Germany), and the blocks trimmed and sectioned to about 5 × 5 × 4 µm size using a microtome (Leica RM2155). The tissue sections were mounted on glass slides using a hot plate (Leica HI1220, Germany) and subsequently treated in the order of 100%, 90%, and 70% ethanol for 2 min each, respectively. Finally, the tissue sections were rinsed in tap water, stained with the Harris’s haematoxylin and eosin (H&E), and examined under a light microscope (Nikon, Tokyo, Japan).

### 2.5. TUNEL Assay

The samples sections were deparaffinised by immersing slides in fresh xylene in a coplin jar for 5 min at room temperature. This step was repeated once. The slides were then processed according to the manufacturer’s instruction (Promega, Madison, WI, USA). In brief, excess water was drained off and the area surrounding the section wiped with tissue paper. The samples were then flooded with anti-fading solution to the treated sections and finally mounted with glass cover slips. The edges of the cover slips were sealed with clear nail polish and allowed to dry for 10 min. The samples were viewed under confocal microscopy using a standard fluorescent filter set to view the green fluorescence at 520 ± 20 nm and also view red fluorescence of Propidium iodide (PI) at > 620 nm.

### 2.6. Western Blot Analysis

Collected breast tumor samples were subjected to protein extraction. Equal amounts of protein for untreated and citral-treated samples were separated by SDS-PAGE, transferred to nitrocellulose (Millipore, Louis, MO, USA), blocked with 5% nonfat milk, and incubated overnight with primary antibodies against cleaved caspase-3 (ab214430, 17 kDa) (Abcam, Cambridge, UK) and β-actin (ab8227, 42 kDa) (Abcam, Cambridge, UK). The membrane was washed, incubated with HRP-conjugated goat anti-rabbit IgG, washed, and incubated with chemiluminescent detection substrate (Genscript, Nanjing, China). The membrane was quantified by Biospectrum AC ChemiHR 40 system (UVP, Upland, CA, USA) and the band intensity was quantified by the ImageJ software (version 1.46). Relative expression of cleaved caspase-3 between untreated and citral-treated samples was calculated after being normalized to the band intensity of β-actin for each sample. 

### 2.7. Tumor Dissociation and Aldeflour Assay

At the end of treatment period, the mice were humanely euthanized by Ketamine + Xylazine (100 mg/kg:10 mg/kg) and tumors were harvested. Tumor tissues were dissociated mechanically and enzymatically to obtain a single-cell suspension as previously described [16]. Briefly, tumors were minced using scalpel and incubated in medium 199 (Invitrogen, Carlsbad, CA, USA) mixed with collagenase/hyaluronidase (StemCell Technologies, Vancouver, BC, Canada) at 37 °C for 15 to 20 min. The tissues were further dissociated by pipette trituration and then passed through a 40 μm nylon mesh to produce a single-cell suspension. These cells subsequently were subjected to Aldeflour assay and BD FACSCalibur flow cytometer (BD, Franklin Lakes, NJ, USA).

### 2.8. Immunohistochemistry (IHC)

For the IHC assay used to evaluate expression pattern of β-catenin, paraffin sections of 4 µm thickness were baked and prepared according to the procedure. Appropriate positive and negative IHC controls were used under staining run for β-catenin antibody to indicate the presence of any nonspecific binding and false positive results. Cytoplasmic staining was qualified based on intensity of staining as low, moderate, or high. Each scoring parameter (intensity of staining and proportion of cells stained) was analyzed separately.

### 2.9. Secondary BALB/c Mice

Ten secondary BALB/c mice were implanted with tumor cells by injecting the right lower fourth inguinal mammary fat with 100,000 cells harvested from the untreated 4T1 mice group; while the left side were implanted with tumor cells harvested from citral treated 4T1 mice. The growth of tumors was monitored and tumor volumes were measured twice weekly. Mice were humanely euthanized when one of the two tumors reached 300 to 500 mm^3^.

### 2.10. Statistical Analysis

The experiments were carried out at least in triplicates (details of replicates are listed in the figure legend of each result) and results are expressed as mean ± SD. Statistical analysis was done using SPSS version 17.0 (SPSS Inc., IBM, Chicago, IL, USA). Probability values of less than alpha 0.05 (*P <* 0.05) were considered statistically significant.

## 3. Results

### 3.1. Tumor Size and Body Weight of Primary BALB/c Mouse Model

**Figure 1:**
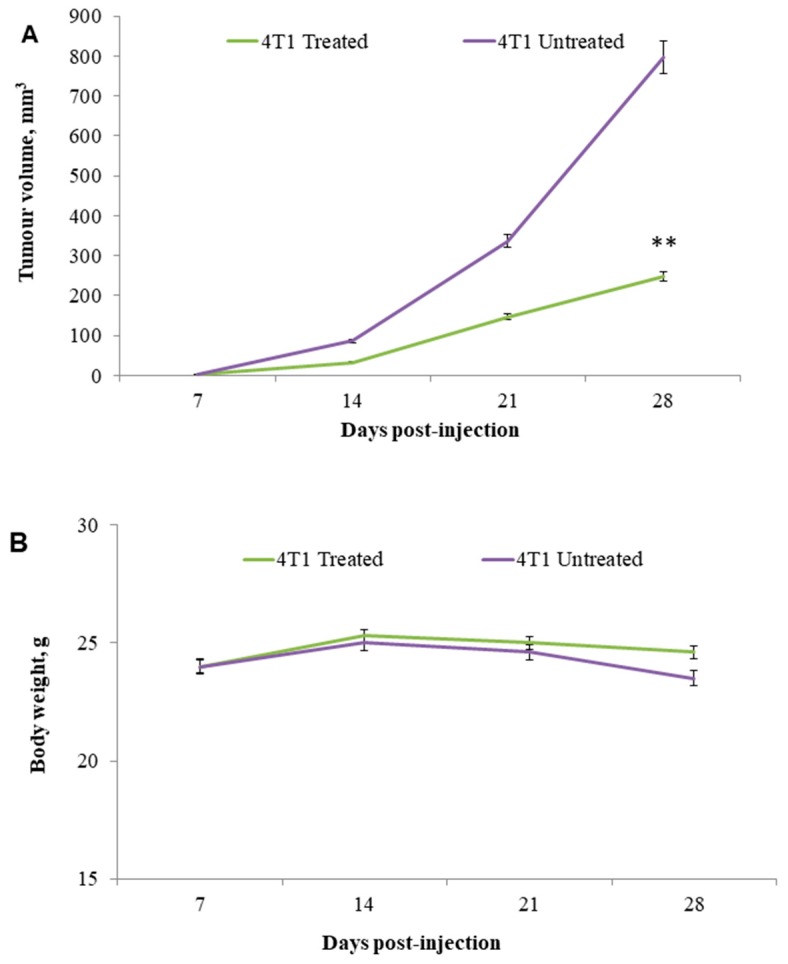
Tumor size in primary xenografts in BALB/c mice. Citral decreased tumor size in primary breast cancer xenografts. BALB/c mice bearing 4T1 cells as xenografts were treated with daily oral feeding of control or 50 mg/kg citral for 2 weeks. Panel **A**: Tumor volumes and panel **B**: Mice body weights were determined. Tumors in citral-treated mice were 50% of the size of control animals at the end of drug treatment. Data are presented as mean ± SD (*n* = 6), (mm^3^). (** *P* ˂ 0.01)**.**

To determine whether citral could target breast cancer cell growth in vivo, a xenograft model using 4T1 cells in BALB/c mice was used. In this study, normal mice treated with 50 mg/kg of citral did not record any mortality or signs of toxicity (results not shown). For the antitumor study, tumor size of the citral-treated mice was 50% smaller than untreated control mice after 2 weeks of treatment (*P* < 0.01; Figure 1A), whereas citral had no apparent toxicity as shown by no changes in body weight (Figure 1B).

### 3.2. Histopathology Analysis

**Figure 2:**
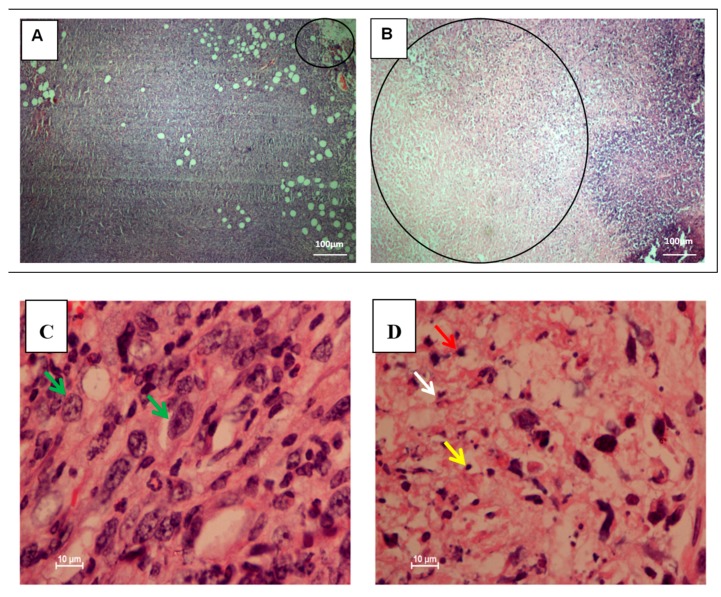
Hematoxylin and eosin (H&E) staining of breast tumors in xenografts of mice induced by 4T1 cells. The untreated 4T1-injected mice breast tumors show focal areas of necrosis/late apoptosis making up about 5%–10% of the lesions (Panel **A**). Treated tumors demonstrated large areas of necrosis/late apoptosis at the central part of the tumors about 70% and about 60% of the lesions, respectively (Panel **B**). Higher magnification of untreated 4T1-injected mice breast tumors show mostly viable tumor cells indicated with pleomorfic vesicular nuclei and hyperchrome prominent nucleoli (green arrow) (Panel **C**). Higher magnification of citral-treated 4T1 breast tumors show large areas of necrosis/late apoptosis indicated with increased number of apoptotic cells (red arrow), cellular debris (white arrow), and nuclear dust (yellow arrow) (Panel **D**), (20×, 100×, scale bar represents 100 and 10 µm in length). The data were generated from six fields per slide, with four slides analyzed from each tumor, and 3 tumors examined from each group. (*n* = 6).

**Figure 3:**
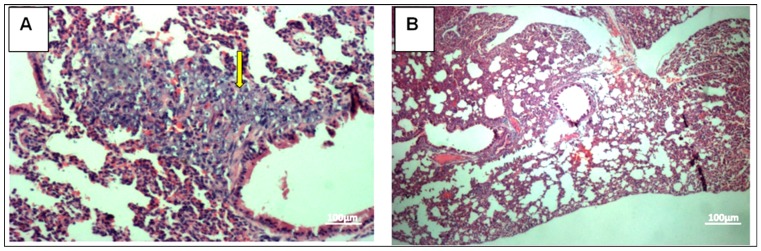
H&E staining of lung tissue in xenografts of mice induced by 4T1 cells. Large malignant cell infiltration was observed in the lung histology of untreated 4T1-injected mice (Yellow arrow). (Panel **A**). Lungs harvested from the treated mice show normal and congested lung tissue with no evidence of tumor (Panel **B**), (20×, bar 10 µm). The data were generated from six fields per slide, with four slides analyzed from each tumor, and 3 tumors examined from each group. (*n* = 6).

To compare the histopathological changes in tumors and lungs of untreated and citral-treated 4T1 challenged mice, breast and lung tissues were harvested, fixed, and stained with hematoxylin and eosin (H&E). The staining showed large areas of necrosis/late apoptosis associated with approximately 60%–70% of lesions, indicated by condensed apoptotic nuclei in the central part of the treated breast tumor of 4T1 injected mice, respectively (Figure 2B). On the other hand, tumors from the untreated groups were observed with mostly viable tumor cells. Patchy and focal areas of necrosis only made up 5%–10% of the lesions in the tumors of 4T1-injected mice (Figure 2A). Higher magnification of untreated 4T1-injected mice breast tumors showed mostly viable tumor cells indicated by pleomorfic vesicular nuclei and hyperchrome prominent nucleoli (Figure 2C), while higher magnification of citral-treated 4T1 breast tumors showed large areas of necrosis/late apoptosis, indicated by an increased number of apoptotic cells, cellular debris, and nuclear dust (Figure 2D). H&E staining for lung tissues showed normal congested lung tissue (Figure 3B), but the untreated group showed infiltration by large malignant cells (Figure 3A).

### 3.3. TUNEL Assay of the Tumor

**Figure 4:**
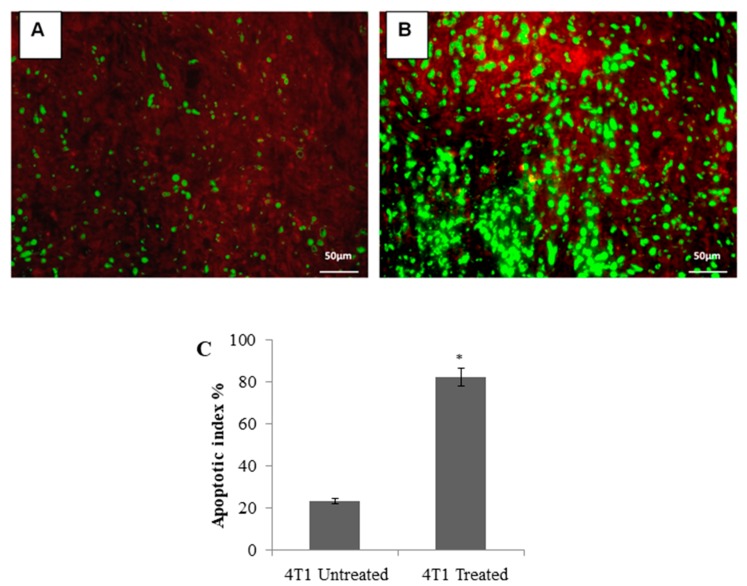
TUNEL assay of breast cancer tissue derived from 4T1 cells. Untreated breast tumor (Panel **A**) compared to treated breast tumor (Panel **B**). Treated breast tumor showed significant apoptosis marked score 4 compared to untreated group. Apoptotic cells (green fluorescence). Nonapoptotic cells (red). Panel **C**: Citral-induced apoptosis in vivo. Each bar represents the apoptosis index expressed as mean ± SD (*n* = 6). Citral treatment caused a significantly higher percentage of TUNNEL-positive apoptotic cells. 20× magnification. The data were generated from six fields per slide, with four slides analyzed from each tumor, and 3 tumors examined from each group. (* *P* ˂ 0.01).

To confirm apoptosis as a mechanism of the tumor growth inhibition, prepared tissue slides were used for TUNEL staining. The breast tumors of the untreated group showed limited (mild–moderate, score 1) apoptosis for 4T1 cells-injected group (Figure 4A). Sections of breast tumors of the citral-treated group showed significant (marked, score 4) (*P <* 0.01) increase in the number of apoptotic cells, evident with higher green fluorescence (Figure 4B).

### 3.4. Western Blot Analysis of Cleaved Caspase 3 of the Tumor

**Figure 5:**
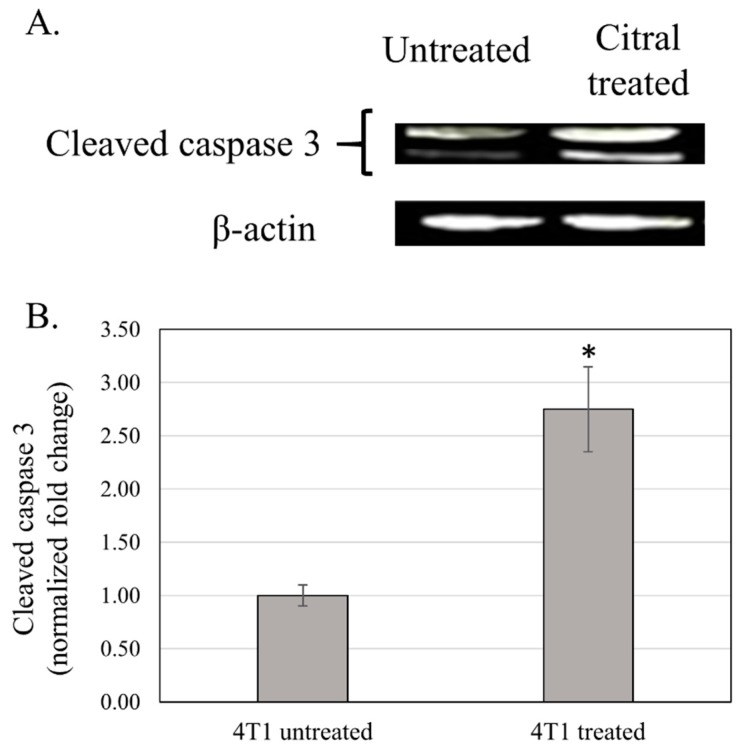
Western blot analysis of cleaved caspase 3 of breast cancer tissue derived from 4T1 cells. Representative blot of cleaved caspase 3 and beta actin of untreated and citral-treated breast tumor (Panel **A**). Panel **B**: Citral-induced cleavage of caspase 3 in the tumor. Each bar represents the normalized fold change of cleaved caspase 3 for untreated and citral-treated tumor as mean ± SD (*n* = 6), (* *P* ˂ 0.01).

Western blot analysis showed that tumor harvested from citral treated mice was detected with 2.75 fold higher level of cleaved caspase 3 comparing to the tumor from untreated mice (Figure 5). 

### 3.5. ALDH Assay of Primary BALB/c Mouse Model

**Figure 6:**
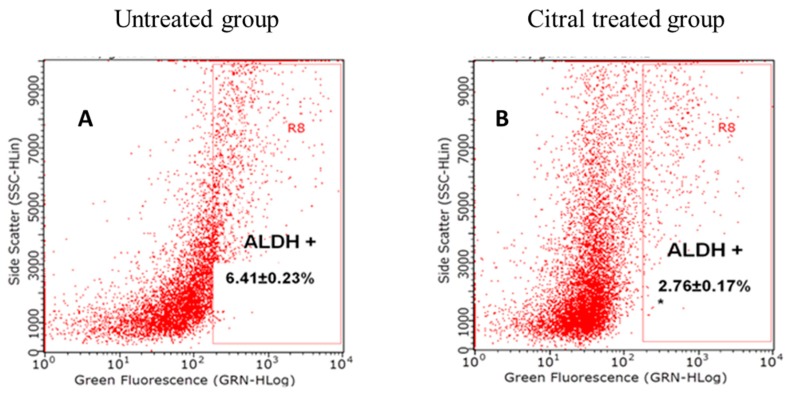
ALDH^+^ cell population in primary breast cancer xenografts induced by 4T1 cells. Flow cytometry analysis of Aldefluor assay showed that citral decreased the ALDH-positive cell population in primary breast cancer xenografts. A set of representative flow cytometry plots is shown in Panel **A**: Control (untreated group). Panel **B**: Citral-treated group. Results for Aldefluor assay are presented as mean ± SD (*n* = 6).

The population of ALDH^+^ tumor cells harvested from the animals were analyzed by Aldefluor assay according to the manufacturer’s instructions. The population of ALDH^+^ cancer cells in 4T1-challenged tumors was reduced by 50% compared to their respective untreated controls, as shown in Figure 6A,B. The decreased ALDH-positive cell population in citral-treated tumors showed that citral was able to reduce the number of ALDH cells, which are responsible for recurrence of tumors in vivo.

### 3.6. Immunohistochemistry of the Tumor

**Figure 7:**
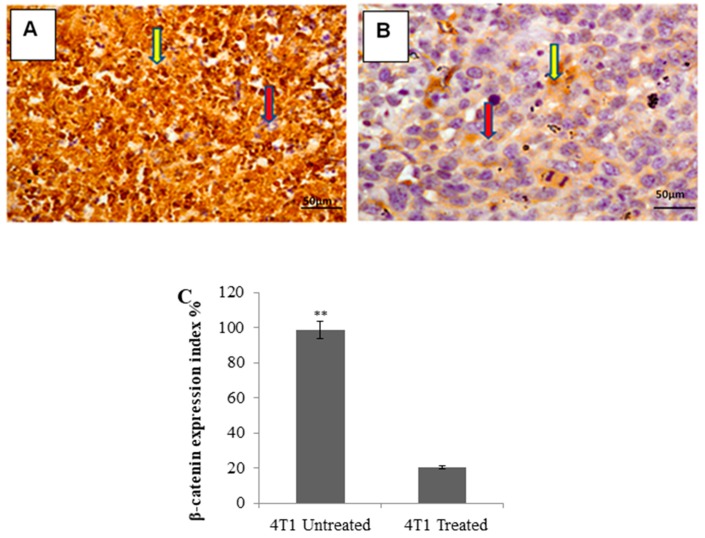
Immunohistochemistry (IHC) staining for β-catenin of breast tumors in 4T1 cells-injected mice. Untreated group (Panel **A**) showing significantly higher expression of the β-catenin marker compared to citral-treated group (Panel **B**) in mice breast tumors (Panel **C)**, each bar represents mean ± SD (*n* = 6). Red arrow: Negative cells, yellow arrow: Positive cells. (IHC) 20× magnification. The data were generated from six fields per slide, with four slides analyzed from each tumor, and 3 tumors examined from each group. (** *P* ˂ 0.01).

Immunohistochemical (IHC) staining showed that in the untreated group, the breast tumors of 4T1 cells-injected mice significantly expressed β-catenin (dark brown color) (Figure 7A), while in the citral-treated group, a significant reduction in β-catenin marker expression in the breast tumors of the injected mice was indicated (Figure 7B).

### 3.7. Secondary BALB/c Mouse Model

**Figure 8:**
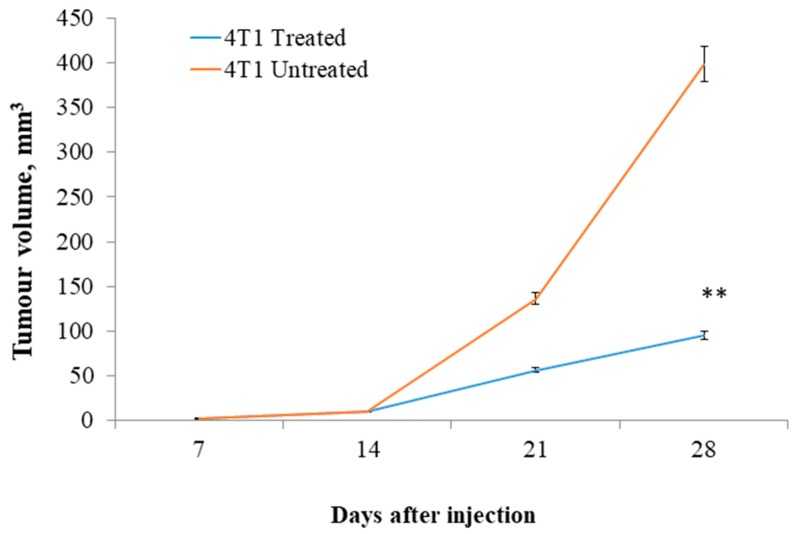
Secondary tumor growth in BALB/c mice. Tumorigenesis of the tumor cells harvested from the citral-treated mice was delayed compared to the tumor cells harvested from the untreated mice when reimplanted in the normal mice (*n* = 6 per group). (** *P* ˂ 0.01).

The ability of residual cancer cells to initiate tumors upon reimplantation in mice is a more definitive assay for showing tumorigenicity of residual tumor cells after treatment. Therefore, the growth of secondary tumors in BALB/c mice inoculated with a primary tumor obtained from primary xenografts of mice were examined. Each recipient mouse was injected with 100,000 cells obtained from citral-treated tumors and control tumors in the inguinal mammary fat pad. The results showed that 4T1 cancer cells from control animals exhibited rapid tumor regrowth, reaching a final tumor size ranging from 380–420 mm^3^ in secondary BALB/c mice, while tumor cells derived from citral-treated mice developed 4-fold smaller tumors than those derived from the untreated group (Figure 8).

## 4. Discussion

One of the main concerns of cancer patients is cancer relapse. Many patients show tumor recurrence and redevelopment of a tumor due to the ability of resistant cancer stem-like cells to initiate a new tumor. Hence, to achieve the best outcome of cancer treatment, therapies that can target both differentiated cancer cells and stemness of cancer cells give the best advantage [17]. Citral, an aldehyde component in *Cymbopogon citrates,* was reported to have cytotoxic and apoptotic-inducing activities [9]. Recent preliminary in vivo studies showed that citral can significantly reduce the tumor size of 4T1-challenged mice [14]. Besides induction of apoptosis [10,11], our previous in vitro study also showed that citral suppressed the capacity to form secondary spheroids of triple-negative breast cancer MDA-MB231 by downregulating ALDH activity and Wnt signaling [12]. However, the mechanism of in vivo anti-breast tumor effect, especially on the regulation of breast cancer tumor-initiating activity, is yet to be determined. Thus, this study was aimed at examining the in vivo anti-tumor effects, particularly on breast cancer cells with ALDH activity and tumor initiating capability. 

The 4T1 mammary carcinoma is a triple-negative transplantable tumor cell line that is highly tumorigenic and invasive and can spontaneously metastasize from the primary tumor in the mammary gland to multiple distant sites, particularly the lung. Also, the progressive spread of 4T1 metastases in BALB/c mice’s draining lymph nodes and other organs very closely mimics human mammary cancer [13]. In this study, untreated mice challenged with 4T1 cells were observed with tumors after seven days of 4T1 injection. Citral did not cause evident toxicity, as specified by body weight at the end of the treatments (Figure 1B). In addition, tumors in mice injected with 4T1 cells were found to have reduced four times the size of tumors in the citral-treated group. In vitro IC_50_ value differs 18-fold between doxorubicin (0.5 µg/mL) [18] and citral (9.0 µg/mL) on 4T1 cells. Compared to the reference [18], reduction of the size of 4T1 tumor by citral was higher than the 5 mg/kg doxorubicin, which reduced 1.7 times the size of 4T1 tumors reported by Lu et al. [18]. Subsequently, to determine if apoptosis was the predominant factor responsible for tumor growth inhibition, prepared tissue samples were used in hematoxylin and eosin (H&E) and TUNEL staining. Induction of apoptosis was confirmed in tumors treated with citral compared to untreated tumors using H&E staining (Figure 2), the presence of TUNEL-positive cells (Figure 4), and higher fold of cleaved caspase 3 (Figure 5). These results support the previous in vitro studies [9,10], which reported that citral induced apoptosis in MDA-MB231 cells. In addition, lung histopathology showed infiltration of malignant cells in the untreated mice, but not the citral-treated mice, indicating that citral treatment prevented the 4T1 metastasis into the lung, supporting the in vitro antiinvasion/migration effect reported by Nordin et al. [10].

Specific ALDH isoenzymes such as ALDH1 not only act as stemness cell markers, but also play important roles in self-protection, differentiation, and expansion, which cause tumor relapse. Thus, ALDH can act as drug-detoxifying enzymes that are responsible for neutralization of therapeutic agents, which have been associated with activation of Wnt/β-catenin signaling, and thus have been proposed as a molecular target in cancer drug discovery [19]. Several natural products, such as proepigallocatechin gallate from green tea [20] and resveratrol from red wine [7], were also reported as inhibitors of ALDH activity in triple-negative breast cancer cells in vitro. As our previous study had proposed that citral suppressed the renewal of triple-negative breast cancer cells via downregulation of ALDH activity and Wnt signalling related genes [12], tumors from the untreated and citral-treated mice were harvested and subjected to ALDH activity and β-catenin detection. Daily gavage of citral for two weeks diminished the population of ALDH-positive tumor cells induced by 4T1 by more than 50% (Figure 6). Citral reduced the ALDH-positive population by >50% compared with that of the control mice (*P* < 0.01). Decreased ALDH-positive cell population in citral-treated mice suggests that citral may be targeting the stemness population of the breast cancer cells. 

Gangopadhyay et al. [21] and Hatsell et al. [22] have reported that overexpression of β-catenin in mouse mammary glands leads to increased hyperplasia and also mammary tumor formation. In addition, Michaelson and Leder [23] demonstrated that elevated levels of β-catenin have been found in many human tumors and elevated levels of β-catenin have also been associated with poor prognoses in human adenocarcinoma of the breast. Decreased β-catenin expression levels using different compounds on different cancer cells in vivo have shown encouraging antitumor effects in mice. For example, Moran et al. [24] and Greenspan et al. [25] demonstrated that carnosol and ibuprofen delayed the progression of xenografted colon tumors in vivo by suppressing expression of β-catenin in the tumor. In addition, Hu et al. [26], Saud et al. [27], and Avtanski et al. [28] demonstrated that sulforaphane, isorhamnetin, and Honokiol decreased β-catenin expression levels in adenomas, HT-29 colon cancer, and breast cancer in mice, which helped to delay the tumor formation. In this study, the expression of β-catenin, which is the key mediator of Wnt signaling pathway that is responsible for cancer cell renewal [29], was evaluated by immunohistochemical staining. The β-catenin immunohistochemical staining (IHC) (Figure 7) analysis showed accumulation of β-catenin in the tumor of the untreated group. On the other hand, the citral-treated group was observed with reduction in the staining of the β-catenin marker. This result proposed that citral may delay the tumor formation by reducing the tumor-initiating capability alongside induction of apoptosis in cancer cells. The ability of tumors to recur and differentiate, as determined by the reimplantation of primary tumor cells in secondary animals, is a more definitive functional assay [30]. Jeong et al. [31] have shown that calcitriol significantly decreased mouse breast tumor-initiating cells and suggested that the inhibition of Wnt/β-catenin pathway is an important mechanism for mediating the tumor-initiating cells’ inhibitory activity in this tumor model. Our results showed that cancer cells from control animals exhibited rapid tumor regrowth, reaching a final tumor size ranging from 380 to 420 mm^3^ in secondary BALB/c mice (Figure 8). Tumors derived from tumor cells harvested from the primary citral-treated group resulted in four times smaller tumor volume compared to the tumors initiated from the primary untreated group. These findings postulate that delayed formation of 4T1 tumors in primary and secondary xenografts of mice may be due to the reduction of β-catenin in citral-treated tumors.

## 5. Conclusions

In this study, citral was able to delay breast cancer tumor formation and lung metastasis in mice by inhibiting the capacity to form secondary tumors and by inducing apoptosis on 4T1 breast cancer cells. Future studies to understand the detailed mechanisms of citral using additional types of cancer cells are needed to support the potential clinical use of citral as an anticancer agent.

## References

[1] Sawant S., Shegokar R. (2014). Cancer research and therapy: Where are we today?. Int. J. Cancer Ther. Oncol..

[2] Larzabal L., El-Nikhely N., Redrado M., Seeger W., Savai R., Calvo A. (2013). Differential Effects of Drugs Targeting Cancer Stem Cell (CSC) and Non-CSC Populations on Lung Primary Tumors and Metastasis. PLoS ONE.

[3] Sharpless N.E., Depinho R.A. (2006). The mighty mouse: Genetically engineered mouse models in cancer drug development. Nat. Rev. Drug Discov..

[4] Kawasaki B.T., Hurt E.M., Mistree T., Farrar W.L. (2008). Targeting Cancer Stem Cells with Phytochemicals. Mol. Interv..

[5] Neto C.C. (2007). Cranberry and its phytochemicals: A review of in vitro anticancer studies. J. Nutr..

[6] Shu L., Cheung K.-L., Khor T.O., Chen C., Kong A.-N. (2010). Phytochemicals: Cancer chemoprevention and suppression of tumor onset and metastasis. Cancer Metastasis Rev..

[7] Fu Y., Chang H., Peng X., Bai Q., Yi L., Zhou Y., Zhu J., Mi M. (2014). Resveratrol Inhibits Breast Cancer Stem-Like Cells and Induces Autophagy via Suppressing Wnt/β-Catenin Signaling Pathway. PLoS ONE.

[8] Kakarala M., Brenner D.E., Korkaya H., Cheng C., Tazi K., Ginestier C., Liu S., Dontu G., Wicha M.S. (2010). Targeting breast stem cells with the cancer preventive compounds curcumin and piperine. Breast Cancer Res. Treat..

[9] Chaouki W., Leger D.Y., Liagre B., Beneytout J.-L., Hmamouchi M. (2009). Citral inhibits cell proliferation and induces apoptosis and cell cycle arrest in MCF-7 cells. Fundam. Clin. Pharmacol..

[10] Nordin N., Yeap S.K., Rahman H.S., Zamberi N.R., Abu N., Mohamad N.E., How C.W., Masarudin M.J., Abdullah R., Alitheen N.B. (2019). In vitro cytotoxicity and anticancer effects of citral nanostructure lipid carrier on MDA-MB-231 human breast cancer cells. Sci. Rep..

[11] Thomas M.L., De Antueno R., Coyle K.M., Sultan M., Cruickshank B.M., Giacomantonio M.A., Giacomantonio C.A., Duncan R., Marcato P. (2016). Citral reduces breast tumor growth by inhibiting the cancer stem cell marker ALDH1A3. Mol. Oncol..

[12] Nigjeh S.E., Yeap S.K., Nordin N., Kamalideghan B., Ky H., Rosli R. (2018). Citral induced apoptosis in MDA-MB-231 spheroid cells. BMC Complement. Altern. Med..

[13] Pulaski B.A., Ostrand-Rosenberg S. (2001). Mouse 4T1 breast tumor model. Curr. Protoc. Immunol..

[14] Zeng S., Kapur A., Patankar M.S., Xiong M.P. (2015). Formulation, Characterization, and Antitumor Properties of Trans- and Cis-Citral in the 4T1 Breast Cancer Xenograft Mouse Model. Pharm. Res..

[15] Luo M., Fan H., Nagy T., Wei H., Wang C., Liu S., Wicha M.S., Guan J.-L. (2009). Mammary epithelial-specific ablation of the focal adhesion kinase suppresses mammary tumorigenesis by affecting mammary cancer stem/progenitor cells. Cancer Res..

[16] Li C., Heidt D.G., Dalerba P., Burant C., Zhang L., Adsay V., Wicha M., Clarke M.F., Simeone D.M., Lepelletier Y. (2007). Identification of Pancreatic Cancer Stem Cells. Cancer Res..

[17] Bao B., Ahmad A., Azmi A.S., Ali S., Sarkar F.H. (2013). Overview of cancer stem cells (CSCs) and mechanisms of their regulation: Implications for cancer therapy. Curr. Protoc. Pharmacol..

[18] Lu J., Zhao W., Huang Y., Liu H., Marquez R., Gibbs R.B., Li J., Venkataramanan R., Xu L., Li S. (2014). Targeted Delivery of Doxorubicin by Folic Acid-Decorated Dual Functional Nanocarrier. Mol. Pharm..

[19] Han L., Shi S., Gong T., Zhang Z., Sun X. (2013). Cancer stem cells: Therapeutic implications and perspectives in cancer therapy. Acta Pharm. Sin. B.

[20] Meeran S.M., Patel S.N., Chan T.-H., Tollefsbol T.O. (2011). A Novel Prodrug of Epigallocatechin-3-gallate: Differential Epigenetic hTERT Repression in Human Breast Cancer Cells. Cancer Prev. Res..

[21] Gangopadhyay S., Nandy A., Hor P., Mukhopadhyay A. (2013). Breast Cancer Stem Cells: A Novel Therapeutic Target. Clin. Breast Cancer.

[22] Hatsell S., Rowlands T., Hiremath M., Cowin P. (2003). β-Catenin and Tcfs in Mammary Development and Cancer. J. Mammary Gland. Boil. Neoplasia.

[23] Michaelson J.S., Leder P. (2001). β-catenin is a downstream effector of Wnt-mediated tumorigenesis in the mammary gland. Oncogene.

[24] Moran A.E., Carothers A.M., Weyant M.J., Redston M., Bertagnolli M.M. (2005). Carnosol inhibits β-catenin tyrosine phosphorylation and prevents adenoma formation in the C57BL/6J/Min/+(Min/+) mouse. Cancer Res..

[25] Greenspan E.J., Madigan J.P., Boardman L.A., Rosenberg D.W. (2011). Ibuprofen inhibits activation of nuclear β-catenin in human colon adenomas and induces the phosphorylation of GSK-3β. Cancer Prev. Res..

[26] Hu R., Khor T.O., Shen G., Jeong W.S., Hebbar V., Chen C., Xu C., Reddy B., Chada K., Kong A.N. (2006). Cancer chemoprevention of intestinal polyposis in ApcMin/+ mice by sulforaphane, a natural product derived from cruciferous vegetable. Carcinogenesis.

[27] Saud S.M., Young M.R., Jones-Hall Y.L., Ileva L., Evbuomwan M.O., Wise J., Colburn N.H., Kim Y.S., Bobe G. (2013). Chemopreventive activity of plant flavonoid isorhamnetin in colorectal cancer is mediated by oncogenic Src and β-catenin. Cancer Res..

[28] Avtanski D.B., Nagalingam A., Kuppusamy P., Bonner M.Y., Arbiser J.L., Saxena N.K., Sharma D. (2015). Honokiol abrogates leptin-induced tumor progression by inhibiting Wnt1-MTA1-β-catenin signaling axis in a microRNA-34a dependent manner. Oncotarget.

[29] Macdonald B.T., Tamai K., He X. (2009). Wnt/β-catenin signaling: Components, mechanisms, and diseases. Dev. Cell.

[30] Visvader J.E. (2009). Keeping abreast of the mammary epithelial hierarchy and breast tumorigenesis. Genes Dev..

[31] Jeong Y., Swami S., Krishnan A.V., Williams J.D., Martin S., Horst R.L., Albertelli M.A., Feldman B.J., Feldman D., Diehn M. (2015). Inhibition of Mouse Breast Tumor Initiating Cells by Calcitriol and Dietary Vitamin D. Mol. Cancer Ther..

